# Spatial distribution of temporal dynamics in anthropogenic fires in miombo savanna woodlands of Tanzania

**DOI:** 10.1186/s13021-015-0029-2

**Published:** 2015-07-30

**Authors:** Beatrice Tarimo, Øystein B Dick, Terje Gobakken, Ørjan Totland

**Affiliations:** 1 Department of Ecology and Natural Resource Management, Norwegian University of Life Sciences, P.O. Box 5003, 1432 Ås, Norway; 2 Department of Geoinformatics, School of Geospatial Sciences and Technology, Ardhi University, P.O. Box 35176, Dar es Salaam, Tanzania; 3 Department of Mathematical Sciences and Technology, Norwegian University of Life Sciences, P.O. Box 5003, 1432 Ås, Norway

**Keywords:** Burned area, Carbon stocks, Fire history, Frequency-size distribution, Landsat, Miombo woodland, MODIS, Surface fires

## Abstract

**Background:**

Anthropogenic uses of fire play a key role in regulating fire regimes in African savannas. These fires contribute the highest proportion of the globally burned area, substantial biomass burning emissions and threaten maintenance and enhancement of carbon stocks. An understanding of fire regimes at local scales is required for the estimation and prediction of the contribution of these fires to the global carbon cycle and for fire management. We assessed the spatio-temporal distribution of fires in miombo woodlands of Tanzania, utilizing the MODIS active fire product and Landsat satellite images for the past ~40 years.

**Results:**

Our results show that up to 50.6% of the woodland area is affected by fire each year. An early and a late dry season peak in wetter and drier miombo, respectively, characterize the annual fire season. Wetter miombo areas have higher fire activity within a shorter annual fire season and have shorter return intervals. The fire regime is characterized by small-sized fires, with a higher ratio of small than large burned areas in the frequency-size distribution (β = 2.16 ± 0.04). Large-sized fires are rare, and occur more frequently in drier than in wetter miombo. Both fire prevalence and burned extents have decreased in the past decade. At a large scale, more than half of the woodland area has less than 2 years of fire return intervals, which prevent the occurrence of large intense fires.

**Conclusion:**

The sizes of fires, season of burning and spatial extent of occurrence are generally consistent across time, at the scale of the current analysis. Where traditional use of fire is restricted, a reassessment of fire management strategies may be required, if sustainability of tree cover is a priority. In such cases, there is a need to combine traditional and contemporary fire management practices.

## Background

Anthropogenic fires are historically an integral component of African savannas. They strongly influence the composition, structure and distribution of mesic savannas in particular, where tree cover is not constrained by climatic conditions [1–4]. Fire regimes in African savannas, including the frequency and season of burning, are mainly human regulated [5]. The variability of fire regimes in African savannas is more dependent on human drivers than on climate and thus human drivers may regulate the future of savanna fire regimes under changing climate conditions [6–9]. Human activities associated with fire ignitions and fragmentation of the landscape play a key role in determining the occurrence of fire and resulting spatial extents of burned areas [10–14]. Tropical savannas, predominantly in Africa, contributes the highest proportion of the global burned area [15], and their contribution to biomass burning emissions is substantial [16, 17]. The role of these fires as a management tool or as a threat to woody cover, and in the global carbon cycle, vary within savannas and is dependent on the fire regime. Efforts to change fire regimes in favor of management priorities, such as carbon sequestration, are being challenged in the light of traditional fire regimes that are more suited for the sustainability of savannas [10, 18–21]. Viable fire management plans aiming at maintenance of stored carbon requires an understanding of historical fire regimes at local scales, which is generally lacking for many parts of African savannas. This understanding is required for precise estimates of the contribution of savanna fires in the global carbon dynamics. Characterization of current fire regimes at local scales is required in order to set references against which assessment of changes in burning practices and their contribution to the carbon cycle will be made [22]. This is of particular importance in fire-adapted ecosystems, such as miombo woodlands, that also support a wide range of human subsistence activities.

Fire is regarded essential to the structure and stability of miombo woodlands [23, 24]. Intense fires suppress tree biomass when their frequency is higher than the rate of tree regeneration and growth [23, 25, 26]. Frequent and intense fires threatens the maintenance of stored carbon stocks, and consequently undermines the potential benefits of activities that comprise the reducing emissions from deforestation and forest degradation (REDD+) policy instrument [27]. Fire contributes to long-term degradation that, although significant, has proven difficult to quantify and monitor, and thus receive less attention in REDD+ negotiations compared to deforestation [28–30]. In addition, they impede the enhancement of carbon stocks for REDD+ payments and sustainability of tree cover at large. Tree recruitment and succession are constrained by recurrent fires [23, 31], which instead facilitates grass encroachment and colonization that may fuel more intense and frequent fires [32, 33]. Exclusion of fire on the other hand facilitates tree dominance of the ecosystem [34], which limits the growth of light demanding grasses and consequently fuel loading. The timing of burning further regulates fire effects, such that late dry season fires have adverse effects on both vegetation and soils, whereas prescribed early dry season fires may be a beneficial management tool [23, 26]. Fire management is of crucial importance for successful forest management [23]. However, it is impaired by the limited understanding on which controlled burning treatments are beneficial for respective components of woodland savannas, coupled with the socio-economic dependency from their surroundings, which play a major role in shaping fire regimes. Characterization of the long-term fire regime will contribute to the ongoing efforts to quantify carbon stocks and fluxes for the purposes of monitoring and verification in the context of REDD+ policy framework and for better fire management practices in general.

A key challenge to both the estimation of carbon fluxes from fires and fire management efforts in African savannas is lack of complete and consistent fire records. In Tanzania, the vast majority of fire events stem from anthropogenic ignitions for different purposes, including farm preparation, pasture management, hunting, honey harvesting, charcoal production, arsons, and for security around settlements and roads [35]. Fire records are limited to a few isolated areas that implement fire management plans. In the absence of long term systematic ground fire records, satellite data forms a unique source of the recent fire history [e.g. 36, 37]. Since tropical savanna fires are fueled mainly by grasses and litter, they sweep the ground surface and leave tree crowns and soil sub-surface unaffected. The resulting burned scars persist for a few weeks only [38–41]. Therefore, frequent observations are required to capture most of the area burned in the course of a fire season. Monthly composites of observation of fire events may be representative of the spatial and temporal distribution of African savanna fires [42–44]. Although the use of different satellite systems provides multiple acquisitions every month, data availability is constrained by cloud cover and other limitations.

Datasets on active fires and burned areas derived from along track scanning radiometer (ATSR), SPOT-VEGETATION and moderate resolution imaging spectroradiometer (MODIS), among other satellite sensors, are available in the public domain. They provide fire patterns at a coarse spatial resolution and at very short temporal coverages. However, comparisons of burned areas derived from coarse resolution (1 km) with those derived from finer resolutions (e.g. 30 m) satellites, show that the majority (up to 90%) of small burned areas characteristic of fragmented fires in tropical savannas, are not detected by coarse resolution burned area products [41, 42, 44, 45]. The low detectability of small-burned areas by coarse spatial resolution products limit the efficacy of these products at smaller spatial scales when detailed information is required. There is thus a need to quantify spatial and temporal fire patterns and resulting burned extents at finer resolutions than those available in the public domain.

The availability of Landsat satellite images in the public domain provides the opportunity to extract burned area records since the early 1970s. Methods are being developed for (semi)automatic burned area mapping at finer spatial resolution e.g. [46, 47], which facilitate frequent and complete mapping at local and regional scales. However, few studies have employed the utility of these methods in African savannas. Thus, burned area records are still missing despite the availability of satellite images. We aim at assessing the fire history during the past ~40 years and respective spatial patterns from satellite based data. Burned areas are mapped by fuzzy classification using spectral indices that include infrared wavelengths, since they are more sensitive to fire induced changes than other spectral combinations [48–50]. We discuss the derived fire return intervals, seasonality and burned extents in Tanzanian miombo relative to those from other African savannas, and the observed frequency-size statistics relative to those reported from other ecosystems. We highlight the consistency in the fire regime across spatial and temporal scales and point out priority areas requiring further analyses and reassessment of management practices.

## Results

### Validation of detected burned areas

Table 1 summarizes classification accuracy analysis of detected burned areas. Omissions of burned pixels are mainly in the partially burned areas, which are not included in Table 1.

**Table 1:** Omission and commission errors of burned pixels

Class	Samples		Errors	
	Correctly classified	Incorrectly classified	Omission (%)	Commission (%)
Burned	1,022	365	26.3	0.7
Not burned	7,826	7	0.1	4.5

Kappa coefficient = 0.82.

Based on an independent validation, the overall performance of the fuzzy classification when including partially burned areas was 57%, which is not as good as that of the completely burned areas (Table 1). It should however be noted that the definition of fuzzy membership scores to distinguish burned from partially burned areas (see “Validation of detected burned areas” in the “Methods” section) on one hand, and the subjective element of the result of the visual interpretation on the other hand, might have had an impact on the quantification of the performance of the fuzzy classification.

### Spatial and temporal patterns of burned areas

#### Burned patch sizes

**Fig. 1:**
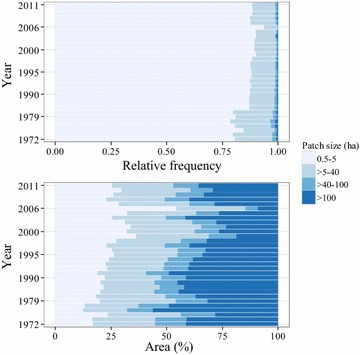
Burned patch sizes in miombo woodlands in Tanzania based on Landsat satellite images. The relative frequency of patch sizes and their corresponding percent contribution to the total area burned during 1972–2011.

For each particular year, the majority (up to the third quartile) of burned patches are less than five hectares in size (Fig. 1). The annual median of burned patch size ranged from 0.8 to 1.4 ha. Small burned patches are more common in wet miombo than in dry miombo areas, with annual median ranging from 0.8 to 1.4 ha and 0.7 to 1.8 ha, respectively. Relatively few and very occasional big fires may reach sizes of up to ~60,000 ha. These account for a large proportion of the total area burned but they tend to decrease in frequency during the 1972–2011 period, relative to other size classes. Overall, small-burned patches, which are a common occurrence over spatial and temporal scales, account for more of the total area burned than large burned patches.

#### Frequency-size distribution of burned patches

Frequency-size statistics of burned areas suggest a fire regime dominated by small-sized fires with scaling, β = 2.16 ± 0.04, with r^2^ = 0.99 for the whole woodland during 1972–2011. Wet miombo has a slightly smaller scaling, β = 2.13 ± 0.03, with r^2^ = 0.99 relative to dry miombo where scaling, β = 2.15 ± 0.04, with r^2^ = 0.99. Given the high number of annual burned patches, it was deemed relevant to analyze the annual frequency-size distributions. Annual analyses resulted in scaling ranging from β = 1.89 ± 0.04 to β = 2.53 ± 0.15, with r^2^ > 0.98 for the whole miombo woodland. Similarly, wet miombo has a slightly smaller scaling than dry miombo from annual analyses, ranging from β = 1.71 ± 0.17 to β = 2.50 ± 0.19, with r^2^ > 0.95 and from β = 1.82 ± 0.05 to β = 2.57 ± 0.43, with r^2^ > 0.94, respectively.

#### Burned extents

**Fig. 2:**
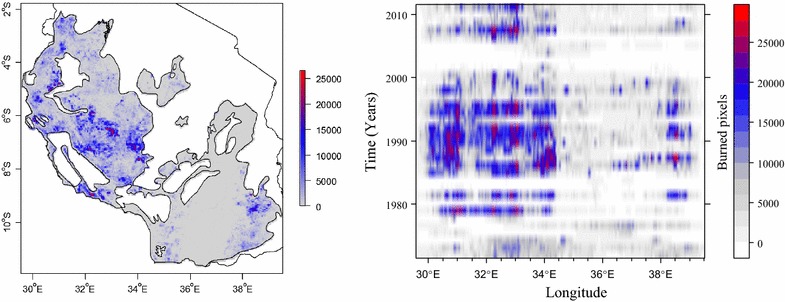
Spatio-temporal patterns of annual burned areas in miombo woodlands in Tanzania. Burned areas from Landsat imagery are summarized at 5 × 5 km resolution, to show the spatial extent for a selected year (1995) on the *left* and spatial–temporal patterns (1972–2011) on the *right*. For the hovmoller diagram on the *right hand side*, *white spaces* represent missing data and areas outside miombo woodland extent while *zero* represents areas not burned.

Figure 2 presents patterns of burned areas detected from Landsat images, summarized at a 5 × 5 km grid. At this scale, fire incidences appear to be consistently within the same spatial extents. Temporal differences in the extent burned per window show an irregular spatial trend. Annually, up to 13.7% and 12.6% of the total area with available imagery was detected as burned in wet and dry miombo, respectively. When combined with partially burned areas, up to 65.8% and 42.1% of wet and dry miombo, respectively, was detected as burned annually. For the whole miombo woodland in Tanzania up to 11.3% is burned annually, while when combined with partially burned areas, up to 50.6% of the woodland is affected by fire annually. Table 2 provides a decadal summary of the contribution of wet and dry miombo areas to the total area burned for the whole woodland. In this table, comparisons are more reliable between dry and wet miombo for the same duration than between durations due to differences in the number of years with available data for each location.

**Table 2:** Burned area characteristics in (a) dry and (b) wet miombo areas in Tanzania during 1972–2011

Duration	P (ha)	A (%)	M [Dry] (%)	RI	Scaling (β)	A_P_
(a) Dry miombo						
1972–1979	930–53,300	3.6–12.6	5.2 [2.9]–11.3 [9.8]	1.6	1.82–2.11	NA
1980–1989	5.4–27,270	3.7–6.5	4.5 [2.2]–8.6 [3.6]	1.8	1.97–2.57	14.3–26.9
1990–1999	1,649–64,650	0.6–8.0	0.8 [0.4]–10.0 [5.1]	2.4	2.00–2.15	10.0–34.1
2000–2011	676.4–21,090	0.7–6.1	1.0 [0.5]–6.6 [4.3]	2.8/3.0^a^	1.93–2.29	4.3–23.6

Duration	P (ha)	A (%)	M [Wet] (%)	RI	Scaling (β)	A_P_
(b) Wet miombo						
1972–1979	1,199–29,050	6.7–11.0	7.6 [2.2]–5.2 [2.3]	1.4	1.83–2.12	NA
1980–1989	8.2–15,760	5.9–11.2	4.5 [2.3]–8.6 [5.0]	1.6	2.04–2.50	16.3–31.3
1990–1999	1,166–36,530	1.6–13.7	0.8 [0.4]–10.0 [4.9]	1.4	2.02–2.14	13.3–52.1
2000–2011	9.1–15,160	0.2–7.7	0.8 [0.1]–6.6 [2.3]	2.0/2.1^a^	1.71–2.26	1.2–25.3

Values in square brackets represents the percent contribution of dry/wet miombo to *M*.

*P* the range of largest annual burned patches detected, may have aggregated with time during the fire season, *A* the range of the total area burned in dry/wet miombo as a percentage of the dry/wet miombo area with data, *A*_*P*_ the total area partially burned in dry/wet miombo as percentage of the total dry/wet miombo area with data, *M* the total area burned in miombo at the same time when *A* was recorded, as percentage of miombo area with data, *RI* Fire return interval observed for every 2,500 ha from Landsat satellite images.

^a^Based on MODIS detected fires for every 314 ha for the period 2001–2013.

### Spatial and temporal patterns of active fires

#### Early and late dry season burning

**Fig. 3:**
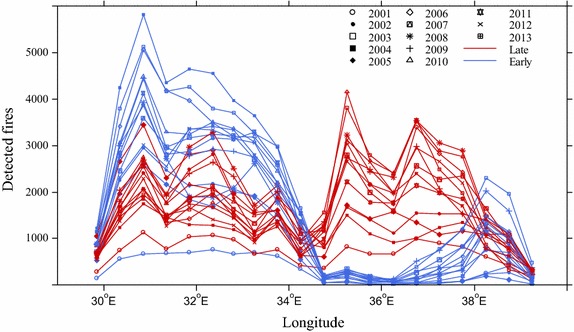
Spatial distribution of MODIS detected fires in miombo woodlands in Tanzania. July was defined to mark the end of early dry season burning for the entire woodland; local variations are likely to occur.

**Fig. 4:**
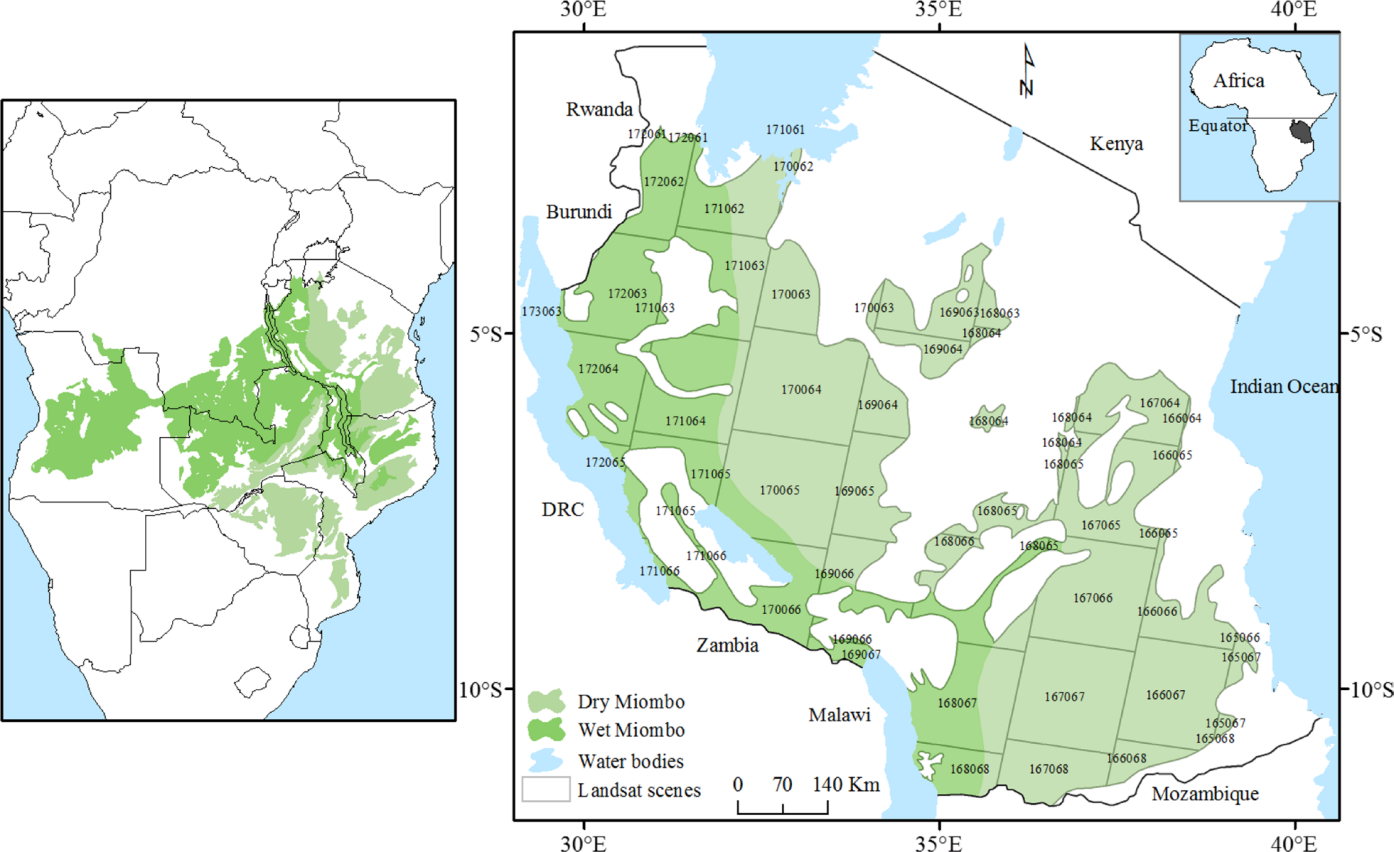
Distribution and classification of miombo woodlands in Tanzania. The map is based on White’s vegetation map of Africa [74]. *Numbers* show the identification (path and row numbers) and extents of Landsat TM/ETM+ scenes.

A west-to-east transition of fire events from early to late burning is observed in Fig. 3. The sudden drop of incidences at ~35.75°E is partly explained by the extent of miombo woodland areas (see Fig. 4) and it marks a distinction between an early dry season burning dominated west to a late dry season burning dominated east.

**Fig. 5:**
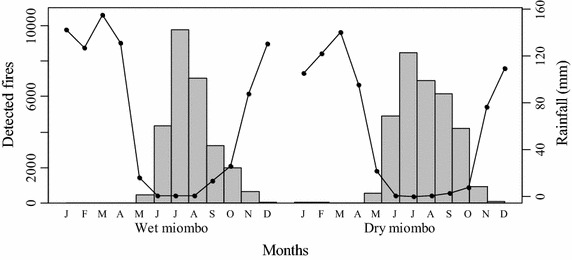
The fire season in wet and dry miombo areas in Tanzania. *Bars* show mean monthly-detected fires for the period 2001–2013 based on MODIS active fires dataset (see “Data sets and preprocessing” section), and *lines* mean monthly rainfall climatology for 1983–2012 period. Rainfall data is sourced from TARCAT [96].

Based on the number of detected fires each month the fire season peaks during the first part of the dry season in July (Fig. 5). To investigate the effect of early dry season burning on late dry season fires, fire radiative power (FRP) values of late dry season fires were compared for those fires which were either close (within 1 km; i.e. approximately within the same fire pixel) or far (>1 km) from early dry season fires during the same fire season. There is no significant reduction, at 95% confidence level, of FRP values in the late dry season fires, which were close to early dry season burned areas than those far from them.

#### Fire activity

**Fig. 6:**
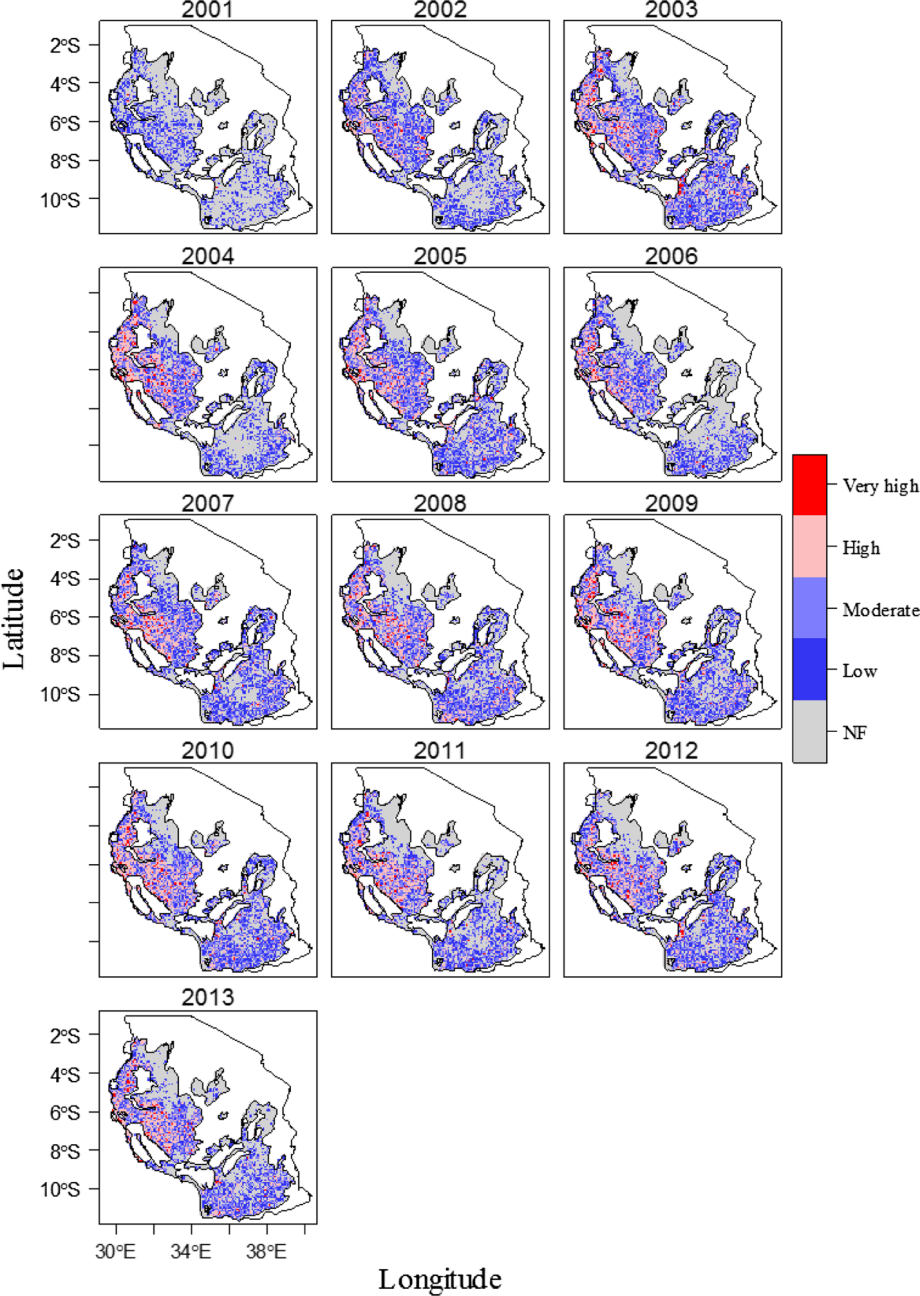
Fire activity in miombo woodlands in Tanzania. Fire activity based on density, proximity and fire radiative power of MODIS detected fire events, and annual length of the fire season, at a 5 × 5 km resolution. *NF* represents areas with no fire activity.

The combined characteristics of detected active fires provide a composite estimate of fire activity given in Fig. 6. Fire activity is consistently high in the western part of the woodland with the exception of areas along its northeastern border. An increasing systematic westward reduction in fire activity is observed along this border during 2001–2013 (Fig. 6). This reduction is associated with the expansion of croplands when interpreted in the context of the GLC-Share land cover types [51]. On the other hand, there is a shift from high to low fire activity between years on the central, south and eastern parts of the woodland.

### Fire return interval

The mean fire return interval for a circular area of ~314 ha, centered at the location of MODIS detected fires was 2.7 years (range 1–13 years) between 2001 and 2013. When the analysis was performed for every 2,500 ha during 1972–2011, based on burned areas detected from Landsat images, the interval was reduced to 2.1 years.

## Discussion

Historical fire regimes are best reconstructed from long-term consistent ground records, charcoal deposition in soils or fire scars on trees with annual growth rings [52–55]. In the absence of these, fire history in miombo woodlands of Tanzania was documented from Landsat satellite images and MODIS detected active fires for the past ~40 years. Both fire prevalence and burned extents have recently decreased (Table 2). This decrease is likely an outcome of a number of contributing factors, including a reduction in miombo woodland coverage through e.g. conversion of the woodland into permanent cultivated fields and fire management practices in some parts of the woodland. Burned areas and detected active fire events are consistently within the same spatial coverage (Figs. 2, 6), at the scale of the current analysis. The lack of an independent burned area perimeter for validation restricted our analysis to burned pixels with the highest confidence, which underestimate the total area burned. When thoroughly validated, an analysis including partially burned areas might increase fire activity and shorten fire return intervals in some parts of the woodland. This however, will not affect the general patterns presented in this study.

### Fire prevalence

About 46% of the woodland area had a mean fire return interval of <2 years for every 314 ha during 2001–2013 period. Field observations in a dry miombo site have shown a mean fire return interval of 1.6 years in Zambia [23], while a return interval of 3 years on a regional scale was observed based on satellite data [24]. In a global study, the fire return interval for African savannas and grasslands has been reduced, from 4.8 years early in 1900 to 3.6 years towards 2000 [15]. Fire return interval and burning seasonality have selective effects on different components of the woodland, as they influence the intensity of fires and extents burned. Results from a study that combined field observations and modelling, from miombo sites in Zimbabwe and Mozambique, show that at least 2 years are required between successive low intensity burns to allow tree establishment and development [25]. Although spatial variation is expected within the scale at which fire activity and return intervals are estimated in the present study, our results indicate that almost half of the woodland is ignited at a return interval that threaten the longer-term sustainability of the tree cover. However, the resulting burned patterns (see “Burned extents” section) indicates an offset, to some extent, of the effect of recurrent fires. About 74% of the woodland area had a mean return interval of <2 years for every 2,500 ha between 1972 and 2011. Therefore, frequent fires have been part of some portions of the woodland for the past ~40 years, when satellite data is available. It is important to note that there is a seasonal and inter-annual variation of burned patches within each of the 2,500 ha and thus the return interval varies at smaller scales. Shorter fire return intervals are observed in wet miombo (Table 2), which are the same areas where annual fire activity is consistently high (Fig. 6). Within dry miombo, shorter return intervals persist in western- as compared to eastern- dry miombo areas. The mean fire return interval was 2.5 and 3.8 years for western- and eastern- dry miombo areas, respectively, for the period 2001–2013. On wider spatial and temporal extents, western- and eastern- dry miombo areas have 1.8 and 2.9 years of fire return intervals, respectively, for the period 1972–2011. Western parts of the woodland, including both dry and wet miombo areas, have higher fire prevalence than eastern parts of the woodland, which consist mainly of dry miombo areas. The higher fire prevalence is mainly a result of the interacting effects of rainfall patterns that influence fuel availability, and ignition sources. In southern African savannas, shorter return intervals occurred in higher rainfall areas but interacted with soil properties and herbivory, over a time period encompassing fire suppression, natural fires and controlled fires [55]. Similarly, in western African savannas, higher fire prevalence occurred in relation to increasing rainfall but interacted with both vegetation type and choices by herders and farmers to burn at different times during the fire season [11]. Rainfall influences productivity of grasses that make up the fuel load, but the fire prevalence is ultimately dependent on human influences on ignitions, fire season and extents burned [6, 7, 11, 56].

The west to east dominance of early and late dry season burning, respectively (Fig. 3), might be explained by differences in the length of the dry season. Parts of the western side of the study area receives light rains during September, from north and extending southward. These light rains continues through the main rainy season, thus reducing the length of the fire season. Central and eastern parts have a unimodal rainfall pattern and thus remain relatively dry until the beginning of another season in November/December, facilitating conditions favorable for late dry season fires.

The observed reduction in fire activity from north towards west (Fig. 6) is associated with expansion of croplands. The expansion of croplands in this area is likely a response to growing mining activities and respectively settlements in the Geita and Kahama districts, north of the study area. Expansion of croplands has had similar effect in northern hemisphere African savannas, where decreasing annual burned area occurred with increasing croplands [57]. As with fire activity, croplands had smaller burned extents when compared to vegetated cover types in the GLC-Share Database [51] for the extent of the study area. Based on GLC-Share cover types, our results show that up to 1.6% of the croplands are burned annually compared to 4.3% of grasslands, 2.6% of tree covered areas, 3.9% of shrubs covered areas and 10% of herbaceous vegetation, aquatic or regularly flooded. The datasets used to compile GLC-Share database for Tanzania is from 2001. Therefore, the values presented above are within a decadal range; from 1995 to 2005.

### Burned extents

#### Burned patch sizes

Small-burned patches, less than five hectares in size, are the most prevalent across spatial and temporal scales. Smaller burned patches are a common occurrence across tropical savannas and are mainly associated with traditional fire management practices [18, 21, 58, 59]. Burned patches with similar sizes were associated with farm preparation in a neighboring Mozambican savanna [60]. Similarly, small fires within African savannas have been associated with agricultural activities and fragmentation of the landscape as a result of high population densities [14, 61]. These fires, burning small patches at a time progressively during the dry season, are generally a desirable management tool and are less damaging to savanna woodlands [18, 62, 63], unless they escape to burn unintended areas. Most of the cases of escaped fires are associated with clearing of new farms as opposed to burning agricultural residues in established agricultural areas. As discussed in the “Fire prevalence” section, our results show that burned extents were smaller in croplands than in other cover types. We could not quantify the effect of other sources of fire on burned patch sizes. However, Butz [21] has observed an increase is large accidental fires and a decrease in small fragmented fires, within a pastoral community in the savannas of northeastern Tanzania. In this area, a decline in nomadic pastoralism has occurred with a trend towards sedentarization and diversified livelihoods [64, 65]. Butz identified changes in rainfall patterns, population growth and fire suppression policies as the drivers of the change in the fire regime. Similar drivers of change in fire regimes persist in western African savannas [8]. In general, competition over land areas increases with a growing population, leading to changes in socioeconomic practices [64] and increasing land fragmentation. Consequently, fire regimes including the frequency, season and sizes of burned areas vary with localized adaptation to these changes in the context of the landscape pattern, and may be influenced by public policies and rainfall patterns [8, 14, 61, 66]. Fires with a higher threat are those ignited within woodland areas where tenure accessibility and private uses are restricted. This threat is associated with increasing homogeneity and buildup of fuels with decreasing human activities [61]. In such cases, the prevailing weather regulates the spread of a fire when ignited [7], as opposed to the human control that fragments the landscape with small fires. In a recent analysis of MODIS burned area product (at 500 m resolution) for the whole of Tanzania between 2000 and 2011, up to 77% of the annual burned area in the country was detected on gazetted land [67]. Although the causes of these fires were not evaluated, it is less likely that they all stem from control burning for fire management purposes. Similarly, protected areas in the southern hemisphere African savannas had relatively larger burned areas than outside protected areas [13]. In the Llanos savannas of Columbia, relatively larger burned areas associated with hunting were observed in a national park compared to indigenous reserves and ranches [68]. In western African savannas, higher densities of fire events occurred in protected areas of Burkina Faso and lower populated areas of Mali [12, 69]. At a smaller scale, the highest tree mortality associated with fire in central Zambia occurred within an encroached part of a national park [70]. This highlights priority fire causes and affected areas that need further detailed analyses and probably a reassessment of management practices. Combining traditional and contemporary fire management practices may achieve reduction in burned extents and consequently biomass burning emissions [71].

We observed a larger scaling of frequency-size distribution, indicating a higher ratio of small relative to large fires, in this study as compared to other ecosystems, e.g. in the United States and Spain [53, 72]. In a global study of fire size distribution, Hantson et al. [14] have also observed a dominance of small fires in our study area, with a similar range of the scaling parameter, β (see “Frequency-size distribution of burned patches” section, Table 2). Small fires are recurrent in both dry and wet miombo areas but dry miombo areas experiences both smaller and larger fires than wet miombo areas (Table 2). The difference in size classes of burned patches in wet and dry miombo contributes to the slightly smaller scaling of frequency-size distribution in wet miombo, which implies large burned patches contribute slightly more to the total area burned in wet than in dry miombo. These large burned patches are partly a result of aggregation of smaller fires during the fire season. Generally, large fires are rare but small fires accumulate to cover extended areas in the course of a fire season each year. Archibald et al. [13] found similar contribution of small fires to the annual burned area in southern hemisphere Africa savannas. Small recurrent fires reduce the risk of occasional large fires, which have recently occurred in areas where fire suppression strategies are enforced.

#### Partially burned areas

Partially burned areas were defined to include intermixed pixels groups that are burned, partially burned and those with a diminishing char signature. They cover relatively wider extents than completely burned areas each year, ranging between 3.2 and 40.6% and 0.8–11.3%, respectively, for the whole woodland. Table 2 provides a decadal summary for wet and dry miombo areas. Between 9 and 14% of Tanzania’s area was detected as burned annually during 2000–2011 from a lower (500 m) resolution burned area product [67]. Of this burned area, 69% occurred in the woodland. Miombo woodland areas covers approximately 90% of the forested areas in Tanzania, implying that much of the burned areas in the country are not detected at the lower resolution. Lower detection rates are possibly higher in the mixed burned–unburned pixels. Similar to completely burned areas, western parts of the woodland have relatively larger extents of partially burned areas, predominantly in wet miombo, than eastern parts of the woodland. Rigorous validation was not performed for partially burned areas, thus they were not further analyzed. However, they provide crucial information for understanding vegetation dynamics, which requires the season and severity of fires at specific areas.

## Conclusions

We have documented the recent fire regime, for the past ~40 years, of the miombo woodland areas of Tanzania at spatial and temporal resolutions that have not been recorded before, to the best of our knowledge. The observed fire patterns for the past 40 years show that the majority of fire events occur in the western parts of miombo woodlands, consisting of wet miombo and western dry miombo areas. Fire events on the western parts of the woodland occur mainly during the first part of the dry season. Thus, an early dry season fire peak characterizes the west while a late dry season fire peak characterizes the east. Almost half of the woodland area has fire return intervals of <2 years. Return intervals are shorter in wet than in dry miombo areas. Short return intervals limit fuel loading and therefore prevents large intense fires. Human activities play a major role in shaping fire regimes. Mainly small sized fires characterize the regime across spatial and temporal scales. Occasional large fires are more frequently detected in dry than in wet miombo areas. Management strategies need to address spatially specific needs of wet and dry miombo areas, in the light of their fire regimes and socio-economic context.

## Methods

### Study area

The study area is miombo woodlands in Tanzania (Fig. 4). Miombo woodlands are disturbance driven moist savannas that are shaped by natural and anthropogenic disturbances, to a larger extent, than by nutrient and water availability [1, 73]. They occur on nutrient poor soils and generally experience a warm-to-hot climate with a dry cold season [24]. The average annual rainfall ranges between 600 and 1,500 mm and falls during 5–6 months [23], followed by an extended dry period. Wet miombo areas, which receives more than 1,000 mm of average annual rainfall, are distinguished from dry miombo areas receiving less than 1,000 mm of average annual rainfall [74]. The woodland is characterized by wooded canopy species and an understory consisting of shrubs and light demanding grass species [24]. The annual production of these flammable, 0.5–2 m tall, grasses every rain season followed by accumulation of litter from the deciduous trees, makes miombo woodland highly susceptible to annual fires.

The fire season extends from the onset of the dry season to its end (Fig. 5), although isolated burning events may occur throughout the year at different localities. During the fire season, individual fires burn small patches at a time with the exception of very occasional big fires in areas where fuel load is accumulated and continuous. Towards the end of the fire season, a mosaic of burned and unburned patches occur.

### Data sets and preprocessing

Landsat Level 1 Terrain (L1T) corrected product satellite images and MODIS collection 5 Level 2 MOD14/MYD14 active fire product form the major data source for this study. We derive fire patterns from the two datasets independently and compare results. Similar patterns will indicate that the datasets are representative of the fire patterns in the study area. This is important because although Landsat provides a finer spatial resolution its temporal resolution (16 days), and further limitation by cloud cover, may limit detection of savanna fires. On the other hand, MODIS detected active fires provide a more complete coverage at high temporal resolution but its temporal coverage is relatively short (since 2000) compared to that of Landsat (since 1972). In addition, MODIS can detect small active fires that are not captured by coarse resolution burned area products [45]. Thus, combining the two datasets benefits from their complementary availability, spatial and temporal characteristics [41].

All available Landsat images were downloaded from the USGS Global Visualization Viewer [75], to cover the study extent (Fig. 4) and for the period 1972–2011. Availability was constrained by image quality, predominantly percentage cloud cover within the study extent. Thus, a complete spatio-temporal dataset was difficult to achieve. For each year, processing and analysis was performed for areas where at least one image was available during the fire season. A total of 1,835 scenes, among them 234 MSS, 1,284 TM and 317 ETM+ SLC-On, were processed. Landsat TM imagery was preferred over MSS imagery for the period when both were available. Each image was converted to at-surface reflectance using the Dark Object Subtraction (DOS) method [76–78].

MODIS active fire data for the whole country were downloaded from the Fire Information for Resource Management System (FIRMS) [79], for the period between November 2000 and December 2013. MOD14/MYD14 provides, among others, coordinates of detected fires (the center of fire pixels at 1 km resolution), their acquisition date and time and respective FRP. Fire locations within miombo were retrieved and categorized as early dry season burning (January–July) or late dry season burning (August–December). Isolated fire events during the wet season were included in respective dry season burning based on the month of their detection. July was chosen to mark the end of early dry season burning for the entire woodland area, consistent with prescribed early burning between May and July in some parts of Tanzania [80–82]. This distinction was made to capture patterns of fire during the dry season, since the timing of burning influences the intensity and spread of a fire and thus its effects, such that fire management through prescribed burning is recommended during early dry season [23].

### Spatial and temporal patterns of burned areas and active fires

**Fig. 7:**
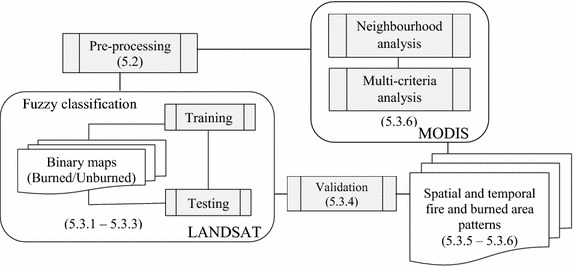
General processing flow with respective detailed sections in *parentheses*.

Burned areas were detected by means of fuzzy classification of spectral indices derived from Landsat satellite images while spatial patterns of active fires were analyzed based on MODIS dataset. We limited our analysis to prevalence, burned extents and spatial patterns of active fires and burned patches. The general processing flow is summarized in Fig. 7. Analyses were performed in GRASS GIS [83] and R version 3.1.0 [84].

#### Training and testing of the fuzzy classification

A total of 523,092 pixels were sampled by visual interpretation from representative scenes for training and testing purposes. Burned areas were identified based on color composites with SWIR, NIR and VIS bands in RGB display, an approach that has been employed to extract image based training, and testing samples [46, 47, 85]. Active fires captured by Landsat satellite images and the analyst’s field experience were utilized in line with the color composites. These formed the basis for selection of spectral indices.

#### Spectral Indices used for fuzzy classification

Spectral indices commonly used for burned area mapping were identified based on literature review. Eleven indices with a potential for discriminating burned areas in sparsely vegetated areas were selected and tested. These included BAI [49], BAIM [86], BAIM_L_ [46], GEMI [87], MIRBI [50], NBR_S_ [46], NBR_L_ [88], NBR_2_ [47], NDVI, SARVI [89] and SAVI [90]. The range of values from individual scenes and the differences among scenes, for which burned pixels were well separated from other cover types, was determined for each index to form the base for fuzzy sets definition (see “Fuzzy membership rules” section). Penalized logistic regression was then employed to analyze the discrimination performance of burned from unburned pixels for each spectral index. An analysis combining sampled pixels from all scenes was also done to investigate how well results at scene level could be generalized.

#### Fuzzy membership rules

Fuzzy discrimination employs membership rules that are defined in terms of fuzzy sets [91], whose elements differentiate definite members from definite non-members and those with some level of uncertainty as to whether they are members or not. Fuzzy classification was experimented for each index individually and for different combinations of indices. Indices and combinations thereof were selected (Table 3) for fuzzy set definition based on how well they distinguished burned from unburned areas. Selected indices conformed to regression results (see “Spectral Indices used for fuzzy classification” section).

**Table 3:** Spectral indices used in fuzzy classification

Indices	Use
BAIM_L_ and MIRBI	Detect burned areas at different post-fire conditions
BAIM_L_ and threshold	Mask bare soil, water^a^, topographic and cloud shadows
NBR_L_ and threshold	Distinguish active fires from other features
BAI	Detect burned areas on MSS imagery

$$BAI = 1/(\rho_{c2} - \rho_{2} )^{2} + (\rho_{c4} - \rho_{4} )^{2} ; \rho_{c2} = 0.1,\rho_{c4} = 0.06 .$$

$$BAIM_{L} = 1/(\rho_{c4} - \rho_{4} )^{2} + (\rho_{c7} - \rho_{7} )^{2} ; \rho_{c4} = 0.05,\rho_{c7} = 0.2 .$$

$$MIRBI = 10\times\rho_{7} - 9.8\times\rho_{5} + 2 .$$

$$NBR = (\rho_{4} - \rho_{7} )/(\rho_{4} + \rho_{7} ) .$$

$$\rho_{ 2} = {\text{Band 2 of MSS on Landsat 4-5 and Band 5 of MSS on Landsat 1-3}} .$$

$$\rho_{4} = {\text{Band 4 of MSS on Landsat 4-5, Band 7 of MSS on Landsat 1-3 and Band 4 of TM/ETM+ }} .$$

$$\rho_{7} = {\text{Band 7 of TM/ETM+ }} .$$

^a^Permanent water bodies were manually masked out from fuzzy classification results.

#### Validation of detected burned areas

**Fig. 8:**
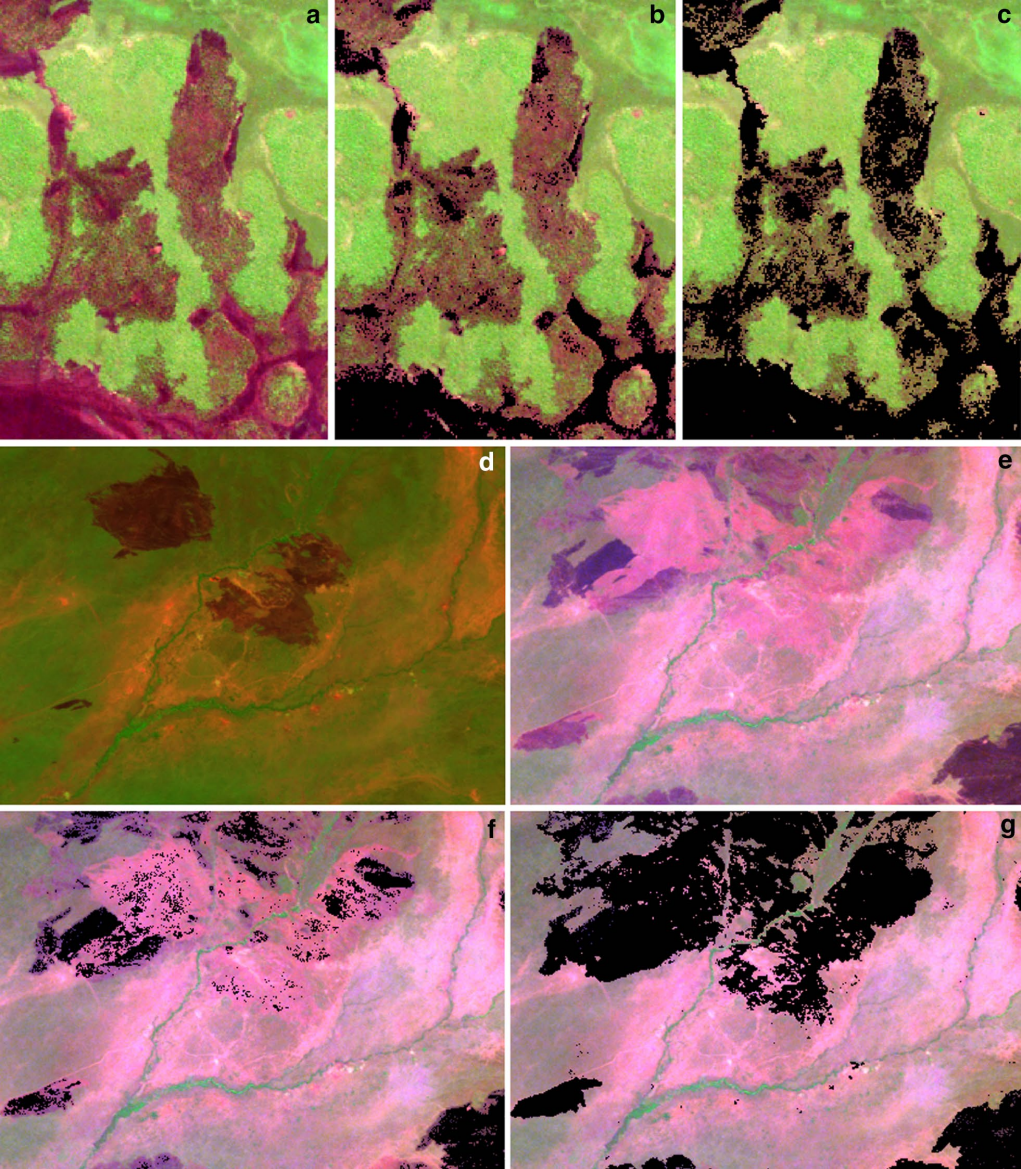
Illustration of areas defined as partially burned. The *top panel* shows an area with mixed burned and unburned pixels (**a**). The *middle panel* shows burned patches at the beginning of the fire season (**d**) and the same area later (**e**) during the fire season. Burned areas (**b**) and (**f**), consist of contiguous groups of burned pixels with a definite fire scar. A mix of burned and unburned pixels and those with a diminishing fire scar were defined as partially burned, shown in (**c**) and (**g**) when combined with burned areas. Detection of burned areas with a diminishing fire scar is desirable when an image from an earlier date during the fire season is not available.

Due to the lack of an independent burned area perimeter for validation, completely burned areas were distinguished from partially burned and unburned areas, based on their membership scores, for the purpose of restricting further analysis to definite burned areas. Partially burned areas consisted of intermixed pixel groups of burned, partially burned and those with a diminishing char signature (Fig. 8). These areas were not included in subsequent analyses but they indicate the spatial and temporal extents of areas affected by fire each year. Validation of completely burned areas, which are referred to as burned areas, was performed based on visual interpretation of randomly selected samples, from another set of representative scenes different from those used for training and testing fuzzy classification. To validate the performance of the fuzzy classification when including also the partially burned areas, we employed visual analysis and unsupervised clustering. This approach, combining visual interpretation and unsupervised clustering, is suitable for discriminating burned areas in African savannas [42]. We adapted the approach described in [47] where three 1,000 × 1,000 pixels image subsets were visually interpreted to delineate burned/partially burned area perimeters. An independent image analyst examined these visually interpreted burned areas with support of false color composites (bands 432 and 741 as RGB) in combination with clustering of the bands 741 data subset, utilizing ERDAS Imagine 2014. The results were then used to validate the combined burned and partially burned area.

#### Burned patch sizes and spatio-temporal variation in burned extents

The sizes of burned patches were calculated based on contiguous burned pixels at scene level, while burned extents from annual mosaics after accounting for multiple detections between acquisitions. The fire return interval based on detected burned areas was determined by overlaying a 5 × 5 km grid on annual burned area maps, thus a return interval for every 2,500 ha. Grid cells containing burned patches >0.5 ha were considered affected by fire in respective years and provided a crude estimate of fire return interval for each cell. We use this estimate for consistency across the spatial and temporal extents with different Landsat data availability and for comparison with MODIS data (see “Occurrence and spatial patterns of active fires” section). The 0.5 ha threshold was selected based on reported burned patch sizes from anthropogenic fire sources in neighboring Mozambican savannas [60]. Frequency-size distributions of burned patches >0.1 and >0.4 ha for TM/ETM+ and MSS imagery, respectively, were examined for the period 1972–2011. Frequency densities of patch size classes were analyzed in log–log space, where the slope coefficient, β, provided the scaling of burned patch sizes i.e. the ratio of the number of large to small fires [53].

#### Occurrence and spatial patterns of active fires

The spatial association of detected fires was examined by Ripley K function for inhomogeneous spatial patterns [92]. Annual fire activity was derived at a 5 × 5 km resolution as a composite measure of active fire characteristics, including density and proximity of fires, annual duration of the fire season and range of FRP values. These combined characteristics of active fires provides a classification of the fire activity that is related to the fire regime [93]. The FRP values, for instance, are associated with the type of vegetation burned [94] and the density of fires is a good predictor of burned areas [13]. The grid resolution was based on both an optimal choice for spatial aggregation when comparing datasets with different resolution as applied in [44, 95] and for practical handling purposes. Fire return interval based on detected active fires was examined by a neighborhood analysis within a 1-km distance from each detected fire. Thus, a return interval for an area of ~314 ha, which is the area of a circle of 1 km radius, centered at the location of detected active fires. This distance was selected to reflect the ground size of detected fire pixels. Results for each year provided fire return interval given locations of detected fires for that year and their average provided mean return interval for all years.

## Abbreviations

BAI: Burned Area Index
BAIM: MODIS Burned Area Index
FIRMS: Fire Information for Resource Management System
FRP: Fire Radiative Power
GEMI: Global Environment Monitoring Index
MIRBI: Mid-Infrared Burn Index
MODIS: Moderate Resolution Imaging Spectroradiometer
NBR: Normalized Burn Ratio
NDVI: Normalized Difference Vegetation Index
SARVI: Soil and Atmospherically Resistant Vegetation Index
SAVI: Soil Adjusted Vegetation Index

## References

[1] Sankaran M, Hanan NP, Scholes RJ, Ratnam J, Augustine DJ, Cade BS (2005). Determinants of woody cover in African savannas. Nature.

[2] Staver AC, Archibald S, Levin S (2011). Tree cover in sub-Saharan Africa: rainfall and fire constrain forest and savanna as alternative stable states. Ecology.

[3] Lehmann CE, Anderson TM, Sankaran M, Higgins SI, Archibald S, Hoffmann WA (2014). Savanna vegetation–fire–climate relationships differ among continents. Science.

[4] Bond W, Woodward F, Midgley G (2005). The global distribution of ecosystems in a world without fire. New Phytol.

[5] Archibald S, Staver AC, Levin SA (2012). Evolution of human-driven fire regimes in Africa. Proc Natl Acad Sci.

[6] Van Der Werf GR, Randerson A, Giglio N, Gobron R, Dolman V (2008). Climate controls on the variability of fires in the tropics and subtropics. Glob Biogeochem Cycles.

[7] Archibald S, Nickless A, Govender N, Scholes R, Lehsten V (2010). Climate and the inter-annual variability of fire in southern Africa: a meta-analysis using long-term field data and satellite-derived burnt area data. Glob Ecol Biogeogr.

[8] Laris P (2013). Integrating land change science and savanna fire models in West Africa. Land.

[9] Archibald S, Lehmann CE, Gómez-Dans JL, Bradstock RA (2013). Defining pyromes and global syndromes of fire regimes. Proc Natl Acad Sci.

[10] Bowman DM, Balch J, Artaxo P, Bond WJ, Cochrane MA, D’Antonio CM (2011). The human dimension of fire regimes on Earth. J Biogeogr.

[11] Mbow C, Nielsen TT, Rasmussen K (2000). Savanna fires in east-central Senegal: distribution patterns, resource management and perceptions. Human Ecol.

[12] Laris P, Caillault S, Dadashi S, Jo A (2015). The human ecology and geography of burning in an unstable savanna environment. J Ethnobiol.

[13] Archibald S, Scholes R, Roy D, Roberts G, Boschetti L (2010). Southern African fire regimes as revealed by remote sensing. Int J Wildland Fire.

[14] Hantson S, Pueyo S, Chuvieco E (2015). Global fire size distribution is driven by human impact and climate. Glob Ecol Biogeogr.

[15] Mouillot F, Field CB (2005). Fire history and the global carbon budget: a 1 × 1 fire history reconstruction for the 20th century. Glob Change Biol.

[16] Andreae MO, Atlas E, Cachier H, Cofer WR, Harris GW, Helas G (1996). Trace gas and aerosol emissions from savanna fires. Biomass Burn Glob Change.

[17] van der Werf GR, Randerson JT, Giglio L, Collatz G, Mu M, Kasibhatla PS (2010). Global fire emissions and the contribution of deforestation, savanna, forest, agricultural, and peat fires (1997–2009). Atmos Chem Phys.

[18] Bird RB, Codding BF, Kauhanen PG, Bird DW (2012). Aboriginal hunting buffers climate-driven fire-size variability in Australia’s spinifex grasslands. Proc Natl Acad Sci.

[19] Moussa K, Bassett T, Nkem J (eds) (2011) Changing fire regimes in the Cote d’Ivoire savanna: implications for greenhouse emissions and carbon sequestration. In: Sustainable Forest Management in Africa: some solutions to natural forest management problems in Africa. Proceedings of the sustainable forest management in Africa Symposium. Stellenbosch, 3–7 November 2008, 2011, Stellenbosch University, Stellenbosch, South Africa

[20] Laris P, Wardell DA (2006). Good, bad or ‘necessary evil’? Reinterpreting the colonial burning experiments in the savanna landscapes of West Africa. Geogr J.

[21] Butz RJ (2009). Traditional fire management: historical fire regimes and land use change in pastoral East Africa. Int J Wildland Fire.

[22] GOFC-GOLD (2014) A sourcebook of methods and procedures for monitoring and reporting anthropogenic greenhouse gas emissions and removals associated with deforestation, gains and losses of carbon stocks in forests remaining forests, and forestation. GOFC-GOLD Report version COP20-1, GOFC-GOLD Land Cover Project Office, Wageningen University, The Netherlands

[23] Chidumayo EN (1997) Miombo ecology and management: an introduction. Intermediate Technology Publications Ltd (ITP), London

[24] Frost P (1996) The ecology of miombo woodlands. In: Campbell BM (ed) The miombo in transition: woodlands and welfare in Africa. CIFOR, Bogor, pp 11–57

[25] Ryan CM, Williams M (2011). How does fire intensity and frequency affect miombo woodland tree populations and biomass?. Ecol Appl.

[26] Trapnell C (1959). Ecological results of woodland burning experiments in northern Rhodesia. J Ecol.

[27] UNFCCC (2011) Report of the conference of the parties on its sixteenth session, held in Cancun from 29 November to 10 December 2010, Adendum, Part Two: action taken by the conference of the parties at its sexteenth session, FCCC/CP/2010/7/Add.1. United Nations Framework Convention on Climate Change, Bonn, Germany

[28] Ahrends A, Burgess ND, Milledge SA, Bulling MT, Fisher B, Smart JC (2010). Predictable waves of sequential forest degradation and biodiversity loss spreading from an African city. Proc Natl Acad Sci.

[29] Barlow J, Parry L, Gardner TA, Ferreira J, Aragão LE, Carmenta R (2012). The critical importance of considering fire in REDD+ programs. Biol Conserv.

[30] Ryan CM, Hill T, Woollen E, Ghee C, Mitchard E, Cassells G (2012). Quantifying small-scale deforestation and forest degradation in African woodlands using radar imagery. Glob Change Biol.

[31] De Michele C, Accatino F, Vezzoli R, Scholes R (2011). Savanna domain in the herbivores-fire parameter space exploiting a tree–grass–soil water dynamic model. J Theor Biol.

[32] Accatino F, De Michele C, Vezzoli R, Donzelli D, Scholes RJ (2010). Tree–grass co-existence in savanna: interactions of rain and fire. J Theor Biol.

[33] Bowman DM, MacDermott HJ, Nichols SC, Murphy BP (2014). A grass–fire cycle eliminates an obligate-seeding tree in a tropical savanna. Ecol Evol.

[34] Bond WJ, Keeley JE (2005). Fire as a global ‘herbivore’: the ecology and evolution of flammable ecosystems. Trends Ecol Evol.

[35] Kikula IS (1986). The influence of fire on the composition of Miombo woodland of SW Tanzania. Oikos.

[36] Giglio L, Randerson J, Van der Werf G, Kasibhatla P, Collatz G, Morton D (2010). Assessing variability and long-term trends in burned area by merging multiple satellite fire products. Biogeosciences.

[37] Russell-Smith J, Ryan PG, Durieu R (1997). A LANDSAT MSS-derived fire history of Kakadu National Park, monsoonal northern Australial, 1980–94: seasonal extent, frequency and patchiness. J Appl Ecol.

[38] Trigg S, Flasse S (2000). Characterizing the spectral-temporal response of burned savannah using in situ spectroradiometry and infrared thermometry. Int J Remote Sens.

[39] Chuvieco E, Opazo S, Sione W, Valle HD, Anaya J, Bella CD (2008). Global burned-land estimation in Latin America using MODIS composite data. Ecol Appl.

[40] Armenteras D, Romero M, Galindo G (2005). Vegetation fire in the savannas of the Llanos Orientales of Colombia. World Resour Rev.

[41] Boschetti L, Roy DP, Justice CO, Humber ML (2015). MODIS–Landsat fusion for large area 30 m burned area mapping. Remote Sens Environ.

[42] Laris PS (2005). Spatiotemporal problems with detecting and mapping mosaic fire regimes with coarse-resolution satellite data in savanna environments. Remote Sens Environ.

[43] Giglio L, Randerson JT, van der Werf GR (2013). Analysis of daily, monthly, and annual burned area using the fourth-generation global fire emissions database (GFED4). J Geophys Res Biogeosci.

[44] Roy DP, Boschetti L (2009). Southern Africa validation of the MODIS, L3JRC, and GlobCarbon burned-area products. IEEE Trans Geosci Remote Sens.

[45] Randerson J, Chen Y, Werf G, Rogers B, Morton D (2012). Global burned area and biomass burning emissions from small fires. J Geophys Res Biogeosci (2005–2012).

[46] Bastarrika A, Chuvieco E, Martín MP (2011). Mapping burned areas from Landsat TM/ETM+ data with a two-phase algorithm: balancing omission and commission errors. Remote Sens Environ.

[47] Stroppiana D, Bordogna G, Carrara P, Boschetti M, Boschetti L, Brivio P (2012). A method for extracting burned areas from Landsat TM/ETM+ images by soft aggregation of multiple Spectral Indices and a region growing algorithm. ISPRS J Photogramm Remote Sens.

[48] Barbosa PM, Grégoire J-M, Pereira JMC (1999). An algorithm for extracting burned areas from time series of AVHRR GAC data applied at a continental scale. Remote Sens Environ.

[49] Chuvieco E, Martin MP, Palacios A (2002). Assessment of different spectral indices in the red-near-infrared spectral domain for burned land discrimination. Int J Remote Sens.

[50] Trigg S, Flasse S (2001). An evaluation of different bi-spectral spaces for discriminating burned shrub-savannah. Int J Remote Sens.

[51] Latham J, Cumani R, Rosati I, Bloise M (2014) FAO global land cover (GLC-SHARE) Beta-Release 1.0 Database, Division LaW

[52] Kasin I, Blanck Y, Storaunet KO, Rolstad J, Ohlson M (2013). The charcoal record in peat and mineral soil across a boreal landscape and possible linkages to climate change and recent fire history. Holocene.

[53] Malamud BD, Millington JD, Perry GL (2005). Characterizing wildfire regimes in the United States. Proc Natl Acad Sci USA.

[54] Scott AC (2000). The Pre-Quaternary history of fire. Palaeogeogr Palaeoclimatol Palaeoecol.

[55] Van Wilgen B, Biggs H, O’regan S, Mare N (2000). Fire history of the savanna ecosystems in the Kruger National Park, South Africa, between 1941 and 1996. S Afr J Sci.

[56] Le Page Y, Oom D, Silva J, Jönsson P, Pereira J (2010). Seasonality of vegetation fires as modified by human action: observing the deviation from eco-climatic fire regimes. Glob Ecol Biogeogr.

[57] Andela N, van der Werf GR (2014). Recent trends in African fires driven by cropland expansion and El Nino to La Nina transition. Nature Climate Change.

[58] Laris P (2002). Burning the seasonal mosaic: preventative burning strategies in the wooded savanna of southern Mali. Human Ecol.

[59] Mistry J, Berardi A, Andrade V, Krahô T, Krahô P, Leonardos O (2005). Indigenous fire management in the cerrado of Brazil: the case of the Krahô of Tocantíns. Human Ecol.

[60] Shaffer LJ (2010). Indigenous fire use to manage savanna landscapes in Southern Mozambique. Fire Ecol.

[61] Archibald S, Roy DP, Wilgen V, Brian W, Scholes RJ (2009). What limits fire? An examination of drivers of burnt area in Southern Africa. Glob Change Biol.

[62] Laris P (2011). Humanizing savanna biogeography: linking human practices with ecological patterns in a frequently burned savanna of southern Mali. Ann Assoc Am Geogr.

[63] Brockett B, Biggs H, Van Wilgen B (2001). A patch mosaic burning system for conservation areas in southern African savannas. Int J Wildland Fire.

[64] McCabe JT, Leslie PW, DeLuca L (2010). Adopting cultivation to remain pastoralists: the diversification of Maasai livelihoods in northern Tanzania. Human Ecol.

[65] Nkedianye D, de Leeuw J, Ogutu JO, Said MY, Saidimu TL, Kifugo SC (2011). Mobility and livestock mortality in communally used pastoral areas: the impact of the 2005–2006 drought on livestock mortality in Maasailand. Pastoralism.

[66] Hudak AT, Fairbanks DH, Brockett BH (2004). Trends in fire patterns in a southern African savanna under alternative land use practices. Agric Ecosyst Environ.

[67] FAO (2013) A fire baseline for Tanzania. Sustainable forest management in a changing climate. FAO-Finland Forestry Programme. Dar es Salaam, Tanzania

[68] Romero-Ruiz M, Etter A, Sarmiento A, Tansey K (2010). Spatial and temporal variability of fires in relation to ecosystems, land tenure and rainfall in savannas of northern South America. Glob Change Biol.

[69] Caillault S, Ballouche A, Delahaye D (2014). Where are the ‘bad fires’ in West African savannas? Rethinking burning management through a space–time analysis in Burkina Faso. Geogr J.

[70] Chidumayo E (2002). Changes in miombo woodland structure under different land tenure and use systems in central Zambia. J Biogeogr.

[71] Russell-Smith J, Cook GD, Cooke PM, Edwards AC, Lendrum M, Meyer C (2013). Managing fire regimes in north Australian savannas: applying Aboriginal approaches to contemporary global problems. Front Ecol Environ.

[72] Moreno M, Malamud B, Chuvieco E (2011). Wildfire frequency–area statistics in Spain. Procedia Environ Sci.

[73] Bond W, Midgley G, Woodward F (2003). What controls South African vegetation-climate or fire?. S Afr J Bot.

[74] White F (1983) The vegetation of Africa: a descriptive memoir to accompany the UNESCO/AETFAT/UNSO vegetation map of Africa by F White. Natural Resources Research Report XX, UNESCO, Paris, France

[75] USGS Global Visualization Viewer. http://glovis.usgs.gov/. Accessed 13 April 2015

[76] Chavez PS (1996). Image-based atmospheric corrections-revisited and improved. Photogramm Eng Remote Sens.

[77] Song C, Woodcock CE, Seto KC, Lenney MP, Macomber SA (2001). Classification and change detection using Landsat TM data: when and how to correct atmospheric effects?. Remote Sens Environ.

[78] Tizado EJ (2013) i.landsat.toar: calculates top-of-atmosphere radiance or reflectance and temperature for Landsat MSS/TM/ETM+/OLI. In: GRASS Development Team (ed) Geographic Resources Analysis Support System (GRASS 7) user’s manual: open source geospatial foundation project. http://grass.osgeo.org

[79] Fire Information for Resource Management System. https://earthdata.nasa.gov/data/near-real-time-data/firms. Accessed 13 April 2015

[80] Nssoko E (2004) Community-based fire management in the Miombo woodlands: a case study from Bukombe District, Shinyanga, Tanzania. Aridlands No 55, May/June 2004

[81] Luoga E, Witkowski E, Balkwill K (2005). Land cover and use changes in relation to the institutional framework and tenure of land and resources in eastern Tanzania miombo woodlands. Environ Dev Sustain.

[82] Hassan SN, Rija AA (2011). Fire history and management as determinant of patch selection by foraging herbivores in western Serengeti, Tanzania. Int J Biodivers Sci Ecosyst Serv Manage.

[83] GRASS Development Team (2012) Geographic Resources Analysis Support System (GRASS) Software. Open Source Geospatial Foundation Project. http://grass.osgeo.org

[84] R Core Team (2014) R: a language and environment for statistical computing. R Foundation for Statistical Computing, Vienna, Austria. http://www.R-project.org/

[85] Koutsias N, Karteris M (2000). Burned area mapping using logistic regression modeling of a single post-fire Landsat-5 Thematic Mapper image. Int J Remote Sens.

[86] Martín M, Gómez I, Chuvieco E (eds) (2005) Performance of a burned-area index (BAIM) for mapping Mediterranean burned scars from MODIS data. In: Proceedings of the 5th international workshop on remote sensing and GIS applications to forest fire management: fire effects assessment. Universidad de Zaragoza, GOFC GOLD, EARSeL, Paris

[87] Pinty B, Verstraete M (1992). GEMI: a non-linear index to monitor global vegetation from satellites. Vegetatio.

[88] Key CH, Benson NC (1999) Measuring and remote sensing of burn severity: the CBI and NBR. Poster Abstract. In: Neuenschwander LF, Ryan KC (eds) Proceedings joint fire science conference and workshop, vol II, Boise, ID, 15–17 June 1999. University of Idaho and International Association of Wildland Fire, p 284

[89] Huete A, Liu H, Batchily K, Van Leeuwen W (1997). A comparison of vegetation indices over a global set of TM images for EOS-MODIS. Remote Sens Environ.

[90] Huete AR (1988). A soil-adjusted vegetation index (SAVI). Remote Sens Environ.

[91] Jasiewicz J (2011). A new GRASS GIS fuzzy inference system for massive data analysis. Comput Geosci.

[92] Baddeley AJ, Møller J, Waagepetersen R (2000). Non-and semi-parametric estimation of interaction in inhomogeneous point patterns. Stat Neerl.

[93] Chuvieco E, Giglio L, Justice C (2008). Global characterization of fire activity: toward defining fire regimes from Earth observation data. Glob Change Biol.

[94] Giglio L, Csiszar I, Justice CO (2006). Global distribution and seasonality of active fires as observed with the terra and aqua moderate resolution imaging spectroradiometer (MODIS) sensors. J Geophys Res Biogeosci (2005–2012).

[95] Eva H, Lambin EF (1998). Remote sensing of biomass burning in tropical regions: sampling issues and multisensor approach. Remote Sens Environ.

[96] Maidment RI, Grimes D, Allan RP, Tarnavsky E, Stringer M, Hewison T (2014). The 30 year TAMSAT African rainfall climatology and time series (TARCAT) data set. J Geophys Res Atmos.

